# **SimGen**: A General Simulation Method for Large Systems

**DOI:** 10.1016/j.jmb.2016.10.011

**Published:** 2017-02-03

**Authors:** William R. Taylor

**Affiliations:** Francis Crick Institute, 1 Midland Road, London NW1 1AT, UK

**Keywords:** CG, coarse-grained, TM, transmembrane, coarse-grained molecular modelling, macromolecular simulation, constraint satisfaction

## Abstract

**SimGen** is a stand-alone computer program that reads a script of commands to represent complex macromolecules, including proteins and nucleic acids, in a structural hierarchy that can then be viewed using an integral graphical viewer or animated through a high-level application programming interface in C++.

Structural levels in the hierarchy range from α-carbon or phosphate backbones through secondary structure to domains, molecules, and multimers with each level represented in an identical data structure that can be manipulated using the application programming interface. Unlike most coarse-grained simulation approaches, the higher-level objects represented in **SimGen** can be soft, allowing the lower-level objects that they contain to interact directly.

The default motion simulated by **SimGen** is a Brownian-like diffusion that can be set to occur across all levels of representation in the hierarchy. Links can also be defined between objects, which, when combined with large high-level random movements, result in an effective search strategy for constraint satisfaction, including structure prediction from predicted pairwise distances.

The implementation of **SimGen** makes use of the hierarchic data structure to avoid unnecessary calculation, especially for collision detection, allowing it to be simultaneously run and viewed on a laptop computer while simulating large systems of over 20,000 objects. It has been used previously to model complex molecular interactions including the motion of a myosin-V dimer “walking” on an actin fibre, RNA stem-loop packing, and the simulation of cell motion and aggregation. Several extensions to this original functionality are described.

## Graphical Abstract

**Figure:**
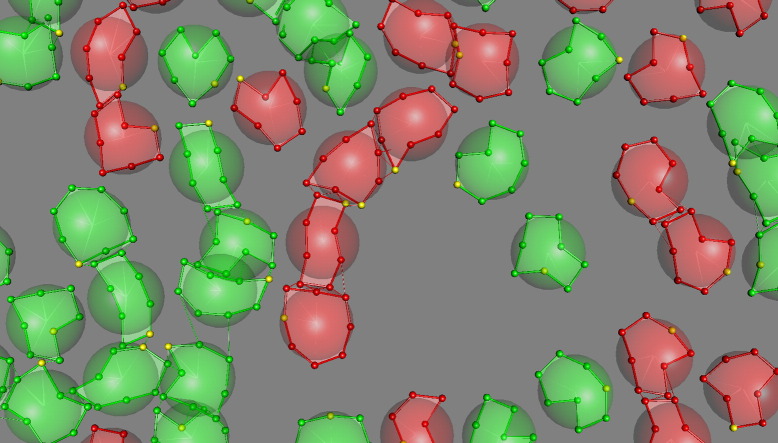

## Introduction

Macromolecular modelling is increasingly being directed towards ever larger molecules and assemblies of molecules, including both nucleic acids and proteins—often in a lipid environment. This trend is driven by a rich harvest of structures, including individual components solved not only by X-ray diffraction and NMR methods but also by new high-resolution electron microscopy. Most importantly, modelling methods are needed to allow this structural information to be extended as widely as possible to interpret the even larger flood of sequence data that has been generated from new rapid sequencing technologies.

Given these data, the range of modelling activity is wide—including the packing of known component protein structures into a complex under the restraint of predicted or experimental pairwise interactions or into a low-resolution envelope provided by electron microscopy or X-ray (see Ref. [1] for a wide-ranging survey). Having obtained a satisfactory model for a complex, the task is often to envisage or predict how it might function, for example, in a molecular motor. Reaching this stage using conventional modelling methods, such as docking and molecular dynamics, can be very time consuming for the investigator and also in the required computer time.

Over a period of many years, methods have been developed to try to reduce both the human and computational burden of constructing and simulating large molecular systems. The most widely adopted approach has been to combine components into groups that then move as a rigid body. This coarse-grained (CG) principle can be applied at any level: a few atoms can be treated as one, a whole amino acid side-chain or nucleotide base can be reduced to a sphere, an alpha-helix or RNA stem loop can be treated as a tube and so on, up to treating a whole domain or protein as a sphere or ellipsoid (for reviews, see Refs. [2], [3], [4], [5]). Ultimately, the greatest saving can be made by treating the whole system as a single sphere, but it should be clear that this results in an unacceptable loss of detail and that any simulation at this level is unlikely to provide biological insight. This tension between resolution and tractability exists to varying degrees across the full range of CG modelling and in an attempt to circumvent it, multi-scale (or hybrid) modelling methods have been developed in which, as the name suggests, multiple levels of representation can co‐exist [6], [7], [8], [9], [10], [11]. The **SimGen** method described here is much simpler than these methods yet incorporates a very general way to include up to several multiple levels of representation without loss of detail at the lowest level.

## Overview

**SimGen** was originally developed just to represent and construct protein molecules as a hierarchy of different structural levels in which each object in the hierarchy can be placed, copied, moved, and linked to construct a complex assembly of one or multiple chains. From this, it was extended to add interaction between objects based on an efficient collision detection algorithm [12], [13]. However, it is not a molecular dynamics program and contains no potentials, forces, or momentum, and its default mode of operation is to do nothing. However, provision is made for a user-supplied routine, called the “driver”, through which every element in the structural hierarchy can be animated using a simple set of geometric operations.

The lowest level object in the hierarchy is a sphere that is usually equated with the α-carbon of the protein chain or nucleotide phosphate. An entry in a user-provided table specifies its properties, such as radius, bond length, and how far two atoms will be pushed apart if their spheres overlap. An equivalent set of properties can be specified for each higher level but includes also the option to be a sphere, ellipsoid, or tube. A parameter is also provided that specifies how much a low-level object (child) should be moved inwards if it lies outside the envelope of its higher-level object (parent). A full description of the various options and parameters can be found in the user manual included in the Supplementary Material.

The simplest use of the program would be to represent an all*-*α-type protein as a string of tubes using their α-carbon coordinate positions from a known PDB structure. As stated above, if the α-carbon radius and the α-helix tube radius are set so that nothing bumps and if all α-carbon atoms are inside their assigned tube, then the model will remain static. However, a default level of random motion can also be specified in the parameter table, which allows the model to explore its local conformational space as a constrained random walk. This motion requires some connection between the levels as it would not make sense to have a parent object wander independently of the children it contains. To do this, the parent is continually moved to the centroid of its children, and conversely, any move to the parent is applied to all its children, and recursively, all their children in turn.

The only other feature of the program that is controlled through a command script (without any need for user programing) is to specify additional links and bonds. This is particularly useful to maintain the local conformation of secondary structure elements, and as it would be tedious to have to specify every virtual hydrogen-bond in a structure, the program automatically assigns virtual bonds between relative α-carbon positions *i* to *i*+3 and *i* to *i*+4 in an α-helix, although the non-local bonds that bind a β-sheet must be specified individually (with the help of a utility program). As links can also be specified between any pair of objects at the same level, a set of restriants can be created, and with the molecule allowed to shake, motions will be directed towards conformations that satisfy the restraints.

In this mode of operation, **SimGen** sounds much like any other constraint satisfaction program. However, the way in which it treats collisions (bumps) between objects is more unique. At the lowest atomic level, this is simply a hard shell repulsion, but at each higher level, two repulsion parameters can be specified, called *hard* and *soft*. When *soft* is zero, objects are repelled using a step size specified by *hard*, but when *hard* is zero, objects are repelled by a step size that is a function of the value of *soft* and the number of children that are in collision between the two parent objects. In other words, objects can either be hard shells or insubstantial containers, or if both parameters are given a value, any combination between—giving the object a “jelly”-like consistency. This is an important feature of **SimGen** as it means that however crude the CG objects are, the details of their contents can still be allowed to interact.

Another unique aspect of **SimGen** that is worth describing briefly is the way in which the different behaviours are implemented. Because there are no potentials or momentum, different actions can be calculated asynchronously, allowing each to be implemented by an independent parallel process (or thread). These have names like bumper, shaker, linker, and keeper, all of which can easily be associated with the actions outlined above. Full details of these routines can be found in the user manual included in the Supplementary Material and also in Ref. [14]. Finally, there is also the viewer that provides real-time visualisation and the driver that must be written by the user if a more purposeful behaviour is required rather than just random (Brownian) movement.

Some details of the method and the way in which it can be animated using a driver application are best illustrated by a series of examples, which will also introduce something of the flavour of the command script used to set up complex models. More details of the algorithms with a particular focus on the implementation of collision detection can be found in Ref. [14].

## Applications

### Actin: a multidomain globular protein

Previously, **SimGen** has been used to simulate the “walking” motion of myosin-V on an actin fibre [15], [16]. As an example of a globular protein hierarchic organisation, the actin molecule from this study will be described to give a flavour of the command language.

**Fig. 1:**
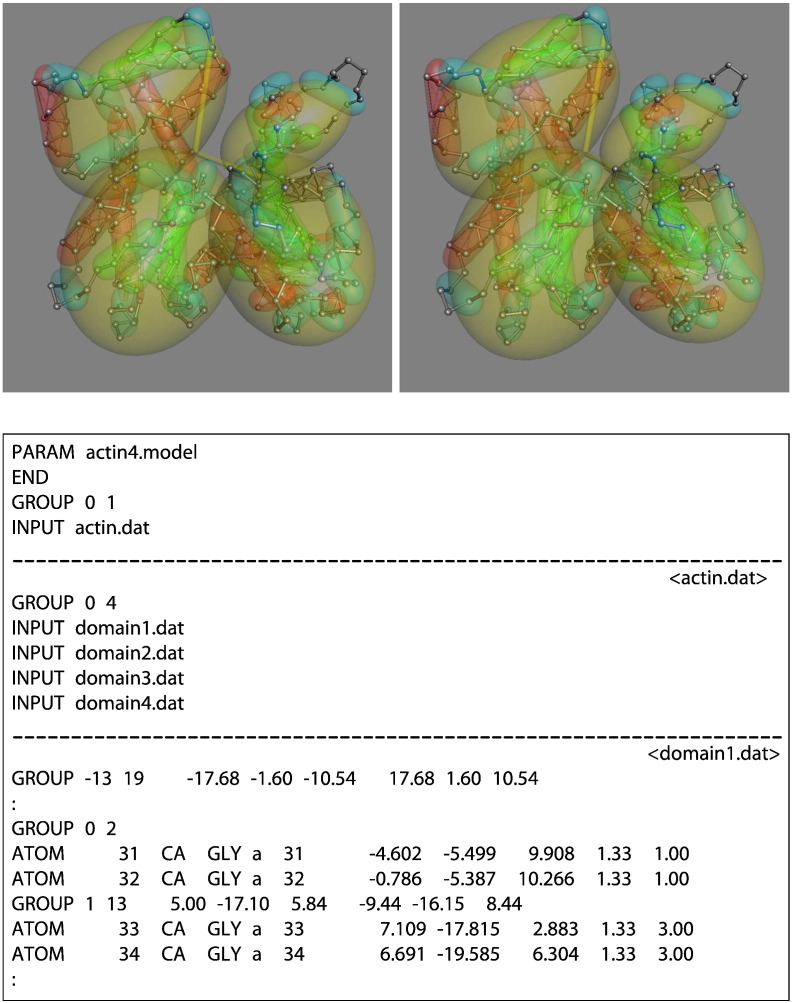
Actin model. The α-carbon backbone trace for actin (PDB code: 1o1g-F) is rendered (in stereo) as a ball-and-stick chain (silver) with virtual hydrogen bonds shown as thin rods. Secondary structure elements are shown as transparent tubes (with hemispherical end-caps), coloured red (α), green (β), and cyan (loops). The four domains are represented by yellow ellipsoids. The script files that created the model are shown in the boxed section, in which the domains are held in separate files that contain the PDB α-carbon records partitioned by GROUP commands. Separate files are divided by a dashed line with the file name in angle brackets to the right.

Levels of structure follow the classic hierarchic organisation of a globular protein as: residue (α-carbon sphere), secondary structure (tube segment), domain (ellipsoid), protein, and multimer. For all bar the residue level, each object in a level is introduced by the command word “GROUP” which can simply be inserted into a standard PDB file. The type of secondary structure is specified by a number 1, 2 for α-helix, β-strands, and 0 for anything else. A tube of an appropriate diameter and length is automatically assigned depending on the structure type, but this can be overridden by additional values on the GROUP command line. Each GROUPed segment at the next higher (domain) level must contain all the α-carbon atoms that belong to the domain whether they are contiguous in sequence or not. If this level has been defined as ellipsoidal, the numbers on the GROUP command can now be used to specify eccentricity and the major axis direction. Both the secondary structure definitions and the domain ellipsoid parameters can be calculated using separate programs based on previous methods [17], [18], [19]. (See Fig. 1)

Additional details of this application with a fuller description of the command files can be found in Ref. [20], and the user manual is included in the Supplementary Material.

#### Rhodopsin: a transmembrane protein

Rhodopsin consists of seven transmembrane (TM) helices arranged in a simple up/down bundle. Each α-helix was modelled as a tube, just as described above for the globular protein, but the seven tubes were then contained in a larger tube rather than an ellipsoid. This tube had a diameter narrow enough to confine the helices in a compact packing arrangement in the plane of the membrane and long enough to allow the helices to shift to a reasonable extent up and down relative to the membrane. Because the ends of the helical tubes are not constrained to lie within their containing tube, they are still free to tilt relative to each other as is commonly observed in such structures (see Supplementary Material Fig. 4a).

This construct for a TM protein was used with both rhodopsin and a variety of proteins of unknown structure in combination with distance restraints derived from an analysis of correlated mutations [21], [22]. Starting from a number of configurations obtained from combinatorial enumeration over a hexagonal lattice [23], the helices were free to move in a random way within the limits of the constraints imposed by the model, and the predicted links between residues were continually refined towards their ideal separation. However, links that remained too long were broken to prevent the disruption of the structure caused by enforcing a combination of mutually inconsistent distances. The resulting models were then ranked on how well they had satisfied the given restraints. The best resulting models were comparable to those obtained using FILM3 or Rosetta [22].

### F_0_-ATPase: animation of a Brownian ratchet

The applications described so far have been either inert (actin) or moving under random perturbations (rhodopsin) and neither have made use of any user code in the driver routine. In this example, a model is constructed for the mitochondrial F_0_-ATPase, which is animated using the driver to simulate a Brownian ratchet.

The F_0_-ATPase consists of a ring of TM helices (C-ring) that rotates past a static component (A-subunit), the structure of which has recently been solved (PDB code: 5ara) [24]. By embedding GROUP commands, the PDB structure can be divided into these components, each of which can then be manipulated directly in the driver routine.

**Fig. 2:**
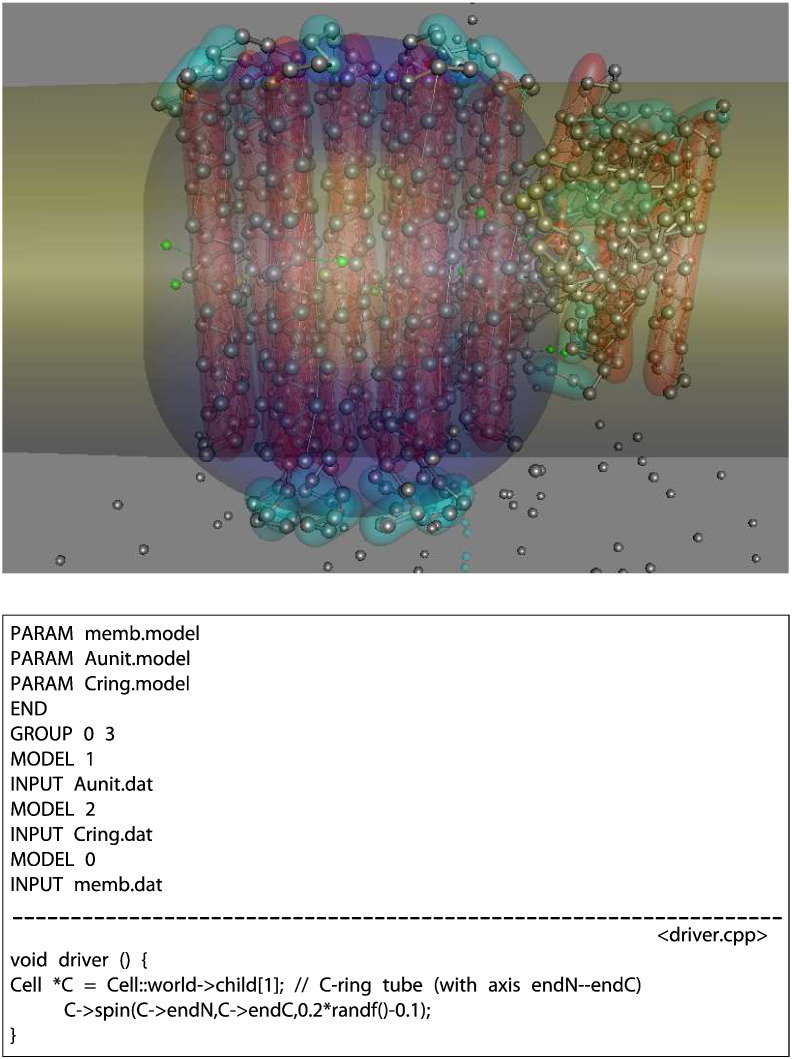
F_0_-ATPase model. The two major membrane components of the mitochondrial ATPase, the A-subunit and C-ring, were set up as separate objects with the latter contained in a tube (dark blue) that can spin on its axis. Both have been located in a large oblate ellipsoid (yellow), part of which is viewed edge-on. Below this is a “swarm” of small spheres representing protons. Protons can attach to selected residues (becoming green spheres on sticks) that allow them to enter the “membrane”. The higher number of protons on one side of the membrane imposes a bias on the direction of the C-ring rotation. In the box, the top-level script for the model is shown that uses three separate parameter models for each component, and below this, a simple driver routine is shown that gives the random motion to the C-ring.

The model setup in Fig. 2 consists of three components, two of which correspond directly to PDB structures and the third is a large oblate ellipsoid (flying saucer) that represents the membrane. This setup script also shows how different models (shapes, sizes, bond lengths, etc.) can be associated with different groups (although all must contain the same number of levels). Neglecting local variable declarations, the driver is a one-line routine that makes the C-ring rotate by random steps around the axis of the tube object that contains the C-ring. (See Fig. 2)

With only a little extra work, small unbonded spheres (representing protons) can be added to the membrane model. These would normally be confined inside their parent container, but this can be reversed in the driver routine to expel them instead. Then, by extending the code in the driver routine, they can be given the ability to form dynamic links to polarisable residues in the protein components, which give them immunity from expulsion from the membrane.

Adding the final condition that any C-ring rotation that takes an unprotonated residue into the membrane is forbidden produces a mechanism that can pick up protons from one side of the membrane, pass them around the C-ring, and release them on the other. If there are equal numbers of “protons” on either side of the membrane, the C-ring moves randomly both clockwise and anticlockwise, but if there is an excess on one side, then the mechanism behaves as a Brownian ratchet giving a bias towards a preferred rotation (the code for this driver routine and a movie of the animation can be found in the Supplementary Material).

#### RNA: “folding” with restraints

**Fig. 3:**
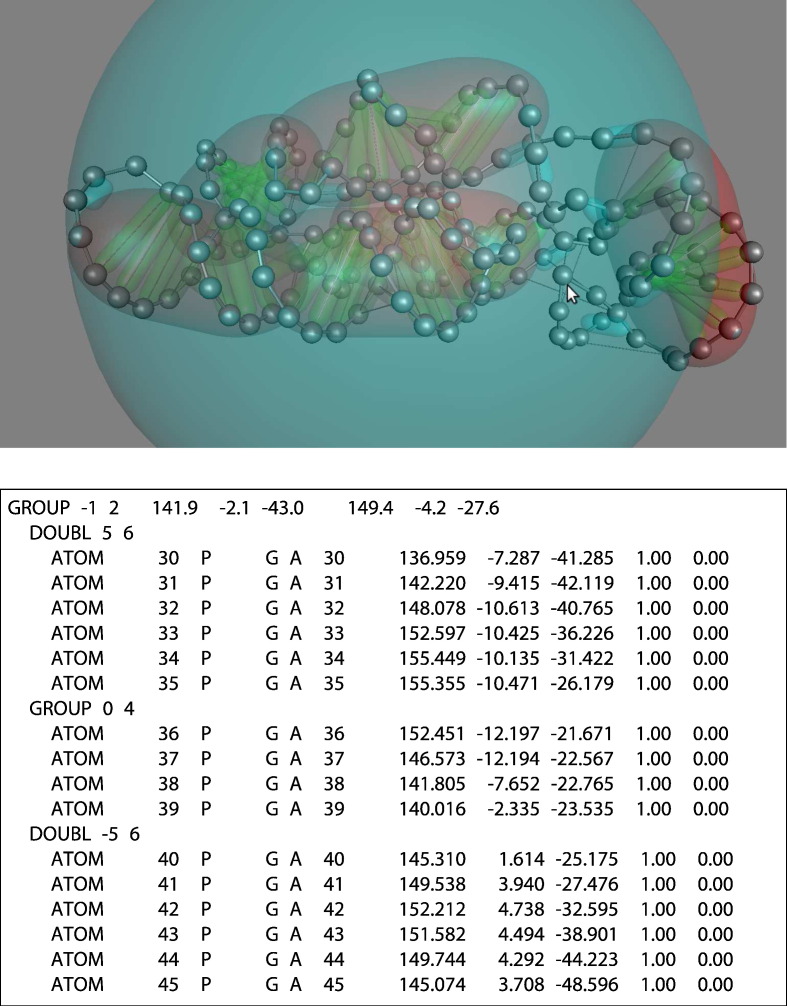
RNA model. The structure of the lysine riboswitch (Rfam code: RF00168) has been set up as a model in which base pairs are represented as green tubes connecting pairs of phosphate atoms (silver spheres). Base pair ladders are then contained in larger red tubes, and the whole molecule is confined in a large sphere. The arrow points to a group of predicted links that form a pseudoknot that has not been defined as a secondary structure. The boxed text shows a fragment of a PDB file containing added commands to define a stem loop. The DOUBL command functions as a GROUP command but instructs the program to look for a matched segment (identified by the 5,-5 pair) to which it will be basepaired, forming a double-stranded secondary structure.

To illustrate the application of the method to nucleic acid molecules, a complex RNA molecule was set‐up to move randomly under a set of imposed pairwise restraints between phosphate atoms (similar to the TM protein model described above). In principle, the hierarchy of structural levels for nucleic acids is similar to that of proteins, including secondary structures and domains, and could be constructed “by hand” using standard commands. However, nucleic acid secondary structures are double stranded, and when represented only by phosphate atoms, it is difficult to keep them in an ideal conformation using only a scaffold of links. To help maintain an ideal geometry, including chirality, the input data can include commands to declare an RNA or DNA molecule and use a special variant of the GROUP command (DOUBL) to identify basepaired segments. (Fig. 3).

#### Cell adhesion and aggregation

The generality of the approach can be seen in an application to simulate cell motion and adhesion on a two-dimensional surface [25]. Cell bodies were modelled as a sphere with a soft (jelly-like) repulsion behaviour that contains a circular ring of hard spheres (referred to as beads rather than atoms to avoid confusion) that can move freely inside and slightly beyond the cell body, consistent with their bond length and bumping constraints (see Supplementary Material Fig. 5).

When any two objects collide (whether cells or molecules), the event is registered in the data structure of each object and can be used in the driver routine to activate a user-defined behaviour, such as a change in speed or direction or the creation of a link between the objects. Different combinations of these behaviours in populations of cells can lead to unexpected “emergent” behaviours that mimic those seen in real cell populations [26] (see Supplementary Material Fig. 6).

In this application, the construction of the model is very simple, but the driver routine is quite extensive, encoding a variety of behaviours that depend on who is linked to whom and how long different links have been maintained. The model remains intrinsically 3D, but the driver also contains a line of code to reduce every Z-coordinate by 1% every cycle. This gentle pull of “gravity” still allows cells to sometimes ride over each other or form a dense “puppy-pile” when they have strong mutual adhesion.

## Implementation

The **SimGen** program is a stand-alone program written in C++ containing its own internal graphics rendering using open-GL (contained in the viewer routine). It is designed to be simultaneously run and viewed on any moderate laptop, but the graphics interface can be “switched-off” for multiple runs, say on a computer cluster. On a laptop, systems containing 20,000–30,000 objects lie around the limit of what can be simulated, which is equivalent to approximately 20 nucleosomes or a myosin-V dimer on a short fibre of 26 actin dimers (modelled as described above). Beyond this, real-time motion becomes “jumpy” with disjointed rendering.

The data structure that holds all the information about an object is the same whatever the level of the object in the hierarchy. This means that the program is scale-free, and every object can be manipulated by the same set of operations. Operations on the hierarchic data structure of grouped objects are processed recursively with the structure of the hierarchy being used to avoid unnecessary computation. For example, if two objects are not bumping, then no checks are made to see if their children are bumping.

Different types of operations, such as moving, bumping, bonding, etc., are performed independently and in parallel with each routine operating on a common data structure. This means that a bump will be repelled even if it results in a bond length being violated, and random moves will always be made no matter what “damage” they do. The situation is similar to a Monte Carlo simulation, in which every move is accepted rather than some being discarded if they lead to restraint violations. However, most operations maintain a list of violations ranked by severity and will try to rectify the worst first, leading quickly to the return of an acceptable conformation.

This relatively unconstrained behaviour can be very useful when exploring a wide conformational space, as a barrier can simply be overcome while the routine that enforces it is momentarily “distracted”. However, it does not mix well with any formulation that uses a potential that requires global evaluation. Whatever time point is chosen to evaluate the potential, there will always be some unresolved conflict that will result in transiently excessive energies. Rather than “freezing” the simulation while these are resolved, an alternative strategy is to have yet another parallel independent routine (or many) that can evaluate conformations “on the fly” and dynamically return corrections to the simulation. From the molecular dynamics viewpoint, this can also be seen as using **SimGen** to enhance conformational sampling and would lend itself well to a Genetic Algorithm implementation [27].

## Conclusions

**SimGen** is primarily intended as a rapid prototyping method in which large macromolecular systems can easily be constructed using a script of commands to place, repeat, bond, and link molecules in a hierarchic structure. The default animation of the resulting system with random Brownian motion can often be all that is required to generate useful behaviour, especially when combined with additional restraints. However, the full potential of the system is only realised with the addition of application-specific code to introduce “purposeful” behaviour of the type that is often seen in molecular motors. For this, the homogeneous data structure that holds the hierarchy of objects provides an intuitive coding interface based on family relationships and high-level geometric operations. To date, the system has only been used to simulate natural molecular complexes and motors, but in the future, it may find a use in the design of artificial systems such as nanomachines.

## Availability

The program, including all source code and scripts, is freely available and can be downloaded†. This site also contains the user manual and the examples used in this work.

## Appendix A Supplementary Data

Supplementary materials. Image 2

## Footnotes

† https://github.com/WillieTaylor/simgen
