# Supplementary material for: SimGen: A General Simulation Method for Large Systems
Source: J Mol Biol. 2017 Feb 3;429(3):408–15. doi: 10.1016/j.jmb.2016.10.011 (PMC5282398; doi:10.1016/j.jmb.2016.10.011)
Supplement: Supplementary file 1 — Supplementary materials. [file mmc1.pdf]

Supplementary Material

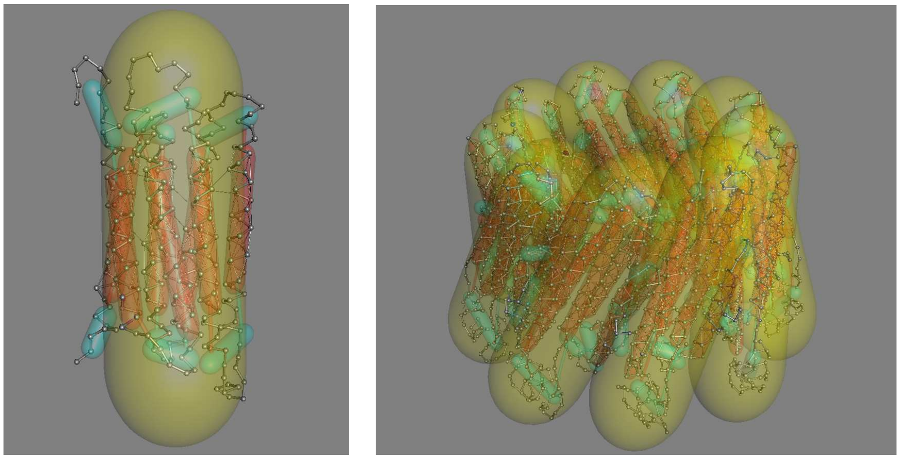

(a) rhodopsin

(b) FlhA

Figure 4: **Transmembrane proteins** were modelled as α-helix tubes inside a "kinder-surprise" confining tube (yellow), the axis of which lies perpendicular to the membrane plane. *a*) A model of rhodopsin with 7-TM helices. *b*) A model of the type-III secretion protein FlhA which is predicted to have 8-TM helices and is thought to form a ring of nine copies in the membrane forming a pore.

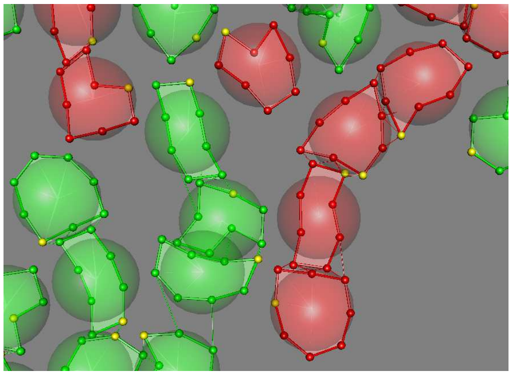

Figure 5: **A simple cell model**. Two types of cell (red and green) have been modelled as a ring of eight beads inside a spherical body. In the model that is shown, each bead can link with any other in a cell of the same colour (seen as fine lines between beads). The yellow bead marks the direction of cell motion (mimicing a leading edge) and when links become over-stretched, they break. All purposeful motion, linking and breaking was implemented in a `driver` routine.

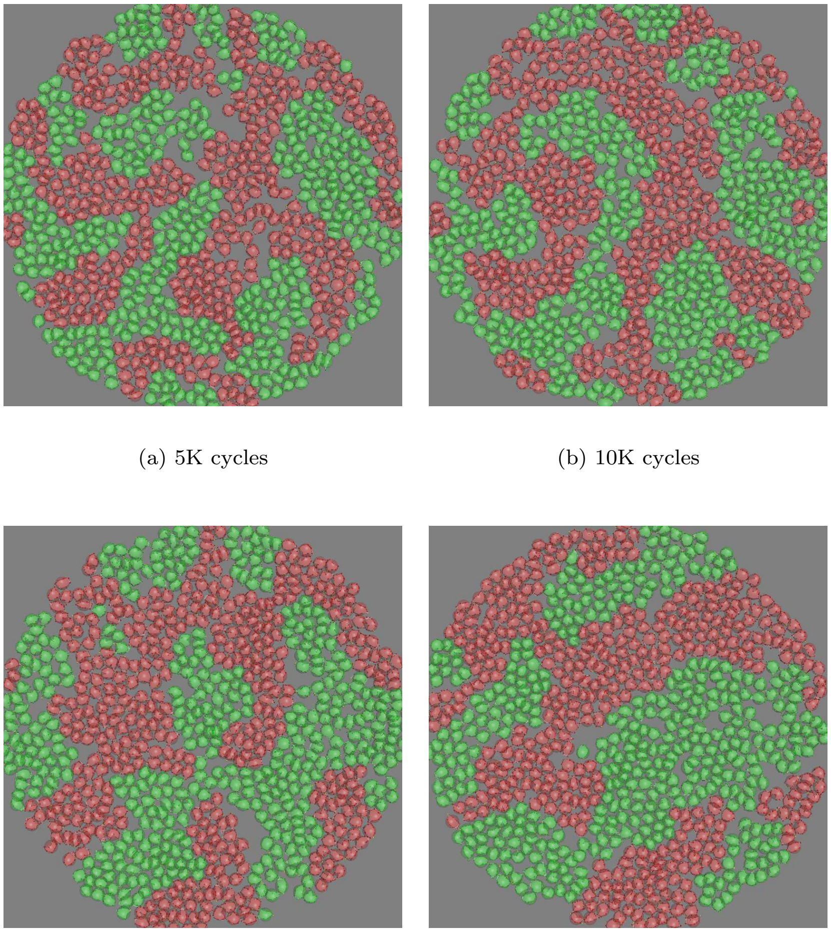

(a) 5K cycles

(b) 10K cycles

(c) 20K cycles

(d) 50K cycles

Figure 6: **Evolution of cell segragation over the course of a simulation**. The model has was set-up to mimic the behaviour of cells interacting through Eph/ephrin signaling.
